# The treatment of vasopressin V2-receptor antagonists in cirrhosis patients with ascites: a meta-analysis of randomized controlled trials

**DOI:** 10.1186/s12876-015-0297-z

**Published:** 2015-06-09

**Authors:** Long Yan, Feng Xie, Jiongjiong Lu, Qingqiang Ni, Changying Shi, Caixi Tang, Jiamei Yang

**Affiliations:** 1 Department of Special Treatment and Liver Transplantation, Eastern Hepatobiliary Surgery Hospital, The Second Military Medical University, 225 Changhai Road, Shanghai, 200438 China; 2 Hepatobiliary and pancreatic surgery center, Zhuzhou Central Hospital, Zhuzhou, 412007 China

**Keywords:** Cirrhosis, Ascites, V2-receptor antagonist, Randomized controlled trials

## Abstract

**Background:**

Ascites is the most common complication of cirrhosis. It may lead to the consequence of poor prognosis and the deterioration of quality of life. Asopressin V2 receptor antagonists is a kind of vaptans, and it has been proved to be effective in hyponatremia patients. We conducted a meta-analysis about treatment of vaptans in cirrhosis patients with ascites.

**Methods:**

Following our selection criteria, we collected a total of 14 studies containing 16 randomized controlled trials (2620 patients) from a series of database about the treatment with vaptans for cirrhosis with ascites patients. The included studies compared the treatment effect of lixivaptan (VPA 985), or RMJ-351647, or satavaptan, or tolvaptan with placebo.

**Results:**

The included vaptans (asopressin V2 receptor antagonists) showed significant effect of increasing the serum sodium concentration for cirrhosis patients (WMD = 2.11 mmol/L, p < 0.00001). Patients also could acquire significant improvement of ascites, as this kind of aquaretics can significantly reduce ascites patients’ weight (WMD = −1.53, p < 0.00001), abdominal girth (WMD = −2.04, p < 0.00001), and the ratio of worsening ascites (RR = 0.51, p = 0.001). Though the drug did not produce more total adverse events (RR = 1.04, p = 0.09) and the total serious events (RR = 1.04, p = 0.42), the emergence of excessive correction of serum sodium concentrations (>145 mmol/L) was more frequently noted in patients under the employment of vaptans (RR = 2.14, 95 % CI [1.45, 3.16], p = 0.0001). Whether with the administration of vaptans for short-term or long-term, no survival benefit was detected from the selected studies.

**Conclusions:**

Asopressin V2 receptor antagonists could play an effective and safe role in symptomatic treatment for cirrhosis patients with ascites, especially for refractory ascites patients who presented insufficient response to conventional diuretics.

## Background

As the most common complication of cirrhosis, ascites is occurred in about 60 % patients within 10 years during the course of compensated cirrhosis [1]. Besides the consequence of poor prognosis, persistent ascites may lead to the development of various subjective and objective symptoms, which could result in deterioration of quality of life (QOL) [2]. Besides the liver transplantation, diuretics also play an important role on the conventional management of ascites, such as aldosterone antagonist (spironolactone) and loop diuretic (furosemide) [3]. But the effect is complicated for the cirrhosis patients due to the varied response to these diuretics [4]. If we administrate diuretics with high dose to achieve a higher response, complications, which are associated with diuretics, could develop more frequently, like electrolyte disturbances, worsening of renal function, hepatic encephalopathy, and so on [5, 6]. On account of these reasons, the development of more effective drugs for the management of ascites is necessary.

The orally and intravenously active non-peptide vasopressin receptor antagonists are called vaptans. Vasopressin V2 receptor antagonists are one of the three kinds of vaptans, and they could competitively bind and block the V2-receptors of arginine vasopressin (AVP) in the renal collecting ducts. So unlike the traditional diuretics, they could induce a highly hypotonic diuresis without affecting the excretion of electrolytes [7]. Previous reports found it was effective to improve the serum sodium concentration of hyponatremia, which was generated from syndrome of inappropriate antidiuretic hormone secretion (SIADH), congestive heart failure patients, and cirrhosis patients with ascites, especially [8, 9]. Some drugs have been approved for the treatment of hyponatremia in Europe [3]. However, some aspects limit the use of vasopressin V2 receptor antagonists. Excessive correction of hyponatraemia, which could be produced by aquaretics, may cause some serious consequence, like osmotic demyelination and myelinolysis; Liver injury is another serious problem people concern with, which is regarded as an important adverse effect from the use of vaptans [10]. In addition to these, there are still some uncertainty and controversies about the effectiveness of vaptans on improvement of the symptoms, prognosis of the cirrhosis patients with ascites. We systematically reviewed the previous randomized controlled trials about the treatment of vasopressin V2 receptor antagonists in cirrhosis patients with ascites.

## Methods

### Search strategy

We searched the studies till February 2014 from the following databases: MEDLINE, PubMed, Embase, the Cochrane Centeral Register of Controlled Trials, Cochrane Database of Systematic Reviews, http://www.ClinicalTrials.gov, and the Chinese BioMedical Literature Database. At the mean time, we check the references of some relevant previous studies to make sure no eligible studies were missed. The searching language was English or Chinese, and the search terms were: vasopressin V2 receptor antagonist, vaptans, mozavaptan, OPC 31260, satavaptan, lixivaptan, VPA 985, RMJ-351647, cirrhosis, liver fibrosis, ascites, hepatic edema, hyponatremia.

### Selection criteria

**Fig. 1:**
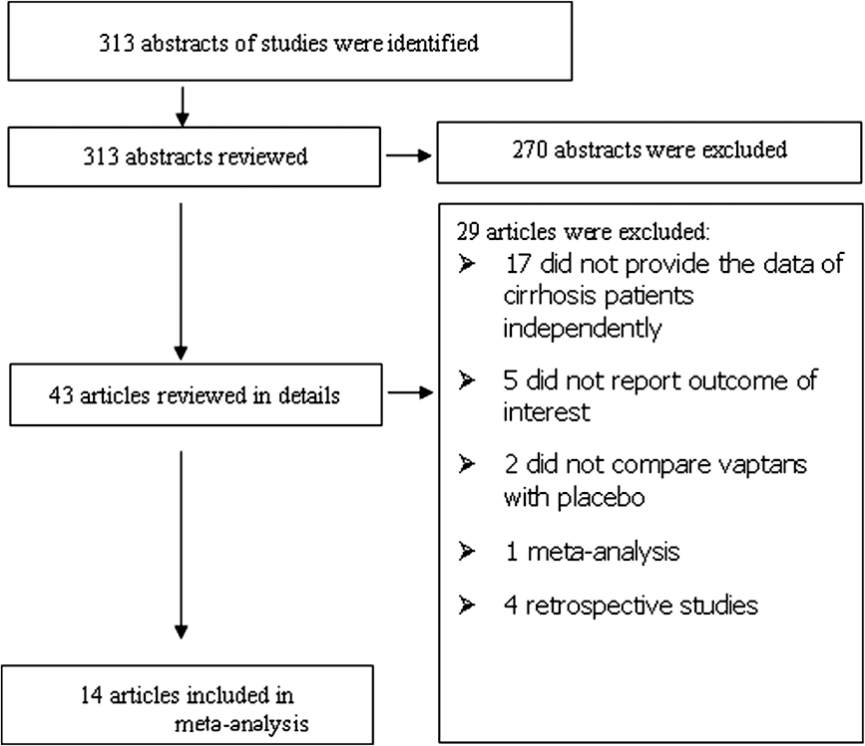
Identification process of the included studies

In our selection process, randomized controlled trials focusing on the treatment for ascites patients with all kinds of of vasopressin V2-receptor antagonist were included (Fig. 1). These adopted vaptans included tolvaptan, conivaptan, lixivaptan (VPA-985), mozavaptan, satavaptan, RMJ-351647. Although conivaptan is antagonists of vasopressin V1A and V2 receptors, it was also included. All patients in the selected studies should be definitely diagnosed as ascites, and the disease of cirrhosis should be the only pathogeny. Whether patients accepted conventional diuretics before or during the study, they would be included.

And the exclusion criteria were: (1) nonhuman studies; (2) non-randomized trials; (3) articles not containing primary data or not stating the data of cirrhosis patients separately; (4) patients with other diseases which also could cause ascites, like SIADH, liver failure, kidney disease; (5) patients with other serious disease, such as neoplastic disease, the end stage of other chronic disease, and severe nervous system disease.

### Data extraction and quality assessment

Two authors independently extracted the data with the tool of Microsoft Office Excel. In this process, the included articles were further checked once more and some studies were excluded for the reason that the data did not meet the inclusion criteria. Following a brief plan which was made previously, the two authors collected the publication details, study characteristics, and the data of outcome assessment. Discrepancies were resolved by discussion until a consensus was made.

The quality assessment of all eligible studies was carried out with the Jadad score (maximum number of points is 5). This evaluation criterion is based on the description of three aspects in the study: random approach, blind method, the withdrawals and dropouts. And each of the first two aspects had totally 2 points and the third had just 1 point [11]. Studies with 3 points or above 3 points were considered with high quality. Sensitivity analysis was presented as performing the fixed effects model and the random effects model meta-analyses at the same time and observing whether the conclusions differed from each other [12]. Publication biases were assessed with funnel plots.

### Outcome Measures and statistical analysis

We assessed the extracted data about the efficacy and safety to synthetically evaluate the treatment of included vaptans for cirrhosis patients with ascites. All included vaptans (tolvaptan, conivaptan, lixivaptan/VPA-985, mozavaptan, satavaptan, RMJ-351647) adopted in these selected studies were regarded as one drug when we analyzed the data. And there just would be one vaptans used in one study.

The primary outcomes measure was survival rate. Secondary outcomes for assessing the efficacy contained the improvement of serum sodium (change of serum sodium concentration, normalized ratio of serum sodium concentration), the improvement of ascites (change of weight and abdominal girth). The occurrence of adverse events, which contained thirsty, excessive correction of serum sodium concentrations and the complications of liver cirrhosis, was assessed to evaluate the safety of vasopressin V2 receptor antagonists for cirrhosis patients with ascites.

Review Manager 5 was mainly used to conduct the statistical analysis, and SPSS 20 was also used if necessary. All of the analysis work was carried out according to the Cochrane Handbook of Systematic Reviews of Interventions [12]. If there were several dose groups comparing with one control group, the data of intervention groups would be gathered up as one to make the comparison of dichotomous variables, and the highest dose group would be regarded as the only drug intervention group when we analyzed continuous variables. Dichotomous variables (normalized ratio of serum sodium level, adverse events, and 1-year survival) were expressed as odds ratios (OR) or risk ratio (RR)with 95 % confidence interval (CI), continuous variables were presented as weighted mean differences (WMD) with 95 % CI. When we pooled the data across studies, fixed-effects model was used in most cases, and random-effects model was adopted when statistical heterogeneity was significant. I2 was calculated in the process of analysis, which was used to assess the statistical heterogeneity [13]. We defined 0-25 % as no or low-level heterogeneity, > = 50 % as significant heterogeneity. When the heterogeneity was significant, we would adopt subgroup to analyze the data and make some explanation, except using random-effects model.

## Results

### Selection of studies

A total of 14 studies containing 16 RCTs and 2620 patients met our selection criteria and were selected [14–27]. Since the study reported by Florence Wong [22] had three trails, all of which separately compared satavaptan with placebo, each trail was considered as an independent RCT when we analyzed the data. Most of selected studies were multicentre. Though 3 studies [25–27] were published as abstract, they were also included for containing important survival data. Among the 16 RCTs, there were 3 comparing lixivaptan (VPA 985) with placebo [14–16], 1 comparing RMJ-351647 with placebo [17], 9 comparing satavaptan with placebo [18–20, 22, 25–27], and 3 comparing tolvaptan with placebo [21, 23, 25]. Conventional diuretics was not used in 2 RCTs [14] [22]. In the rest of selected studies, whether patients in the vaptans arm or in the placebo arm, they would get spironolactone or/and furosemide if doctors considered they had to. Patients adopted sodium-restricted diet in all of the studies if necessary. The basic characteristics of all included studies were presented in Table 1.

**Table 1:** Characteristics of included studies

	Study	Country	Study design	Random method	Blinding	Duration	Conventional diuretic	Treatment	Number of patients	Age (mean ± SD)	Sex (M/F)	Serum Sodium Concentration (mmol/L)
1	Dominique Guyader (2002)	Multicenter trail	RCT	NA	Double-blind	1 d	N	palcebo	5	55.6 ± 10.1	3/2	139.0 ± 3.2
								VPA-985 25 mg	4	58.5 ± 9.5	3/1	140.5 ± 2.1
								VPA-985 50 mg	5	59.4 ± 6.0	5/0	135.7 ± 5.2
								VPA-985 100 mg	4	61.2 ± 8.0	3/1	131.0 ± 6.0
								VPA-985 200 mg	4	62.8 ± 5.8	3/1	139.0 ± 3.0
								VPA-985 300 mg	5	49.0 ± 2.9	4/1	136.0 ± 2.1
2	Alexanderl. Gerbes (2003)	Multicenter trail	RCT	Computerized randomization	Double-blind	7 d	NA	palcebo	20	58 ± 2	15/5	127.3 ± 3.0
								VPA-985 100 mg/d	22	54 ± 3	17/5	128.3 ± 4.1
								VPA-985 200 mg/d	18	56 ± 3	14/4	126.4 ± 4.4
3	Florence Wong (2003)	Multicenter trail	RCT	Computerized randomization	NA	7 d	Y	palcebo	8	NA	NA	127 ± 1
								VPA-985 50 mg/d	8			126 ± 1
								VPA-985 250 mg/d	10			122 ± 2
								VPA-985 500 mg/d	7			125 ± 1
4	PJ THULUVAT H (2006)	USA	RCT	NA	Double-blind	1 d	Y	palcebo	6	50.5 ± 7.6	4/2	136 ± 2
								RWJ-351647 1 mg	6	56.5 ± 5.2	4/2	136 ± 5
								RWJ-351647 2 mg	6	51.5 ± 7	5/1	137 ± 2
								RWJ-351647 5 mg	6	48.3 ± 1.2	5/1	136 ± 3
5	Pere Gines (2008)	Multicenter trail	RCT	Computerized randomization	Double-blind	14 d	Y	palcebo	28	55 ± 10	22/6	NA
								Satavaptan 5 mg/d	28	57 ± 8	16/12	
								Satavaptan 12.5 mg/d	26	56 ± 9	19/7	
								Satavaptan 25 mg/d	28	59 ± 10	20/8	
6	Florence Wong (2010)	Multicenter trail	RCT	Computerized randomization	Double-blind	12 weeks	Y	palcebo	36	60 ± 9	26/10	136 ± 4
								Satavaptan 5 mg/d	39	59 ± 9	32/7	133 ± 6
								Satavaptan 12.5 mg/d	36	59 ± 9	28/8	135 ± 5
								Satavaptan 25 mg/d	40	60 ± 11	29/11	134 ± 5
7	Pere Gines (2010)	Multicenter trail	RCT	Computerized randomization	Double-blind	14 d	Y	palcebo	35	58 ± 9	22⁄13	137 ± 3
								Satavaptan 5 mg/d	40	58 ± 9	30⁄10	137 ± 3
								Satavaptan 12.5 mg/d	35	57 ± 8	26⁄9	136 ± 4
								Satavaptan 25 mg/d	38	55 ± 9	25⁄13	136 ± 2
8	Andres Cardenas (2012)	USA	RCT	Stratified random	Double-blind	30 d	Y	Placebo	57	55 ± 9	38/19	NA
								Tolvaptan 15-60 mg/d	63	52 ± 8	50/13	
9a	Florence Wong (2012)a	Multicenter trail	RCT	Stratified random	Double-blind	52 weeks	Y	placebo	230	56.4 ± 9.2	168/62	NA
								Satavaptan 5 or 10 mg/d	232	56.6 ± 10.2	158/74	
9b	Florence Wong (2012)b	Multicenter trail	RCT	Stratified random	Double-blind	52 weeks	Y	placebo	168	57.0 ± 9.8	113/55	NA
								Satavaptan 5 or 10 mg/d	328	58.9 ± 10.0	237/91	
9c	Florence Wong (2012)c	Multicenter trail	RCT	Stratified random	Double-blind	52 weeks	N	placebo	80	56.2 ± 9.9	50/30	NA
								Satavaptan 5 or 10 mg/d	160	56.4 ± 9.6	108/52	
10	Isao Sakaida (2014)	Japan	RCT	NA	Double-blind	7 d	Y	placebo	80	NA	49/31	135.7 ± 4.1
								tolvaptan 7.5 mg/d	82		52/30	135.3 ± 4.5
11	Kiwamu Okita (2014)	Japan	RCT	NA	Double-blind	7 d	Y	placebo	26	64 ± 10	17/9	NA
								tolvaptan 7.5 mg/d	25	65 ± 9	18/7	
								tolvaptan 15 mg/d	25	65 ± 10	21/4	
								tolvaptan 30 mg/d	25	63 ± 10	15/10	
12	Pere Gines (2007)	Multicenter trail	RCT	NA	Single-blind	52 weeks	Y	placebo	47	NA	NA	132.5
								satavaptan 5 mg/d-50 mg/d	26			131.7
13	Pere Gines (2008)’	Multicenter trail	RCT	NA	Single-blind	52 weeks	Y	placebo	92	NA	NA	NA
								satavaptan 5 mg/d-50 mg/d	47			
14	Florence Wong (2009)	Multicenter trail	RCT	NA	Single-blind	52 weeks	Y	placebo	48	NA	NA	NA
								satavaptan 5 mg/d-50 mg/d	186			

### Quality assessment

When we used Jadad score (maximum number of points is 5) to assess the quality of these selected 16 RCTs (14 studies) basing on their descriptions in the articles, 8 got 5 points [15, 18–22], 5 got 4 points [14, 16, 17, 23, 24], and 3 got 3 points [25–27]. Most of the RCTs were double-blinding (81 %). 50 % of all the 16 RCTs calculated the sample size before the studies.

### Effects of vasopressin V2-receptor antagonist

The effects of vasopressin V2-receptor antagonist for cirrhosis patients with ascites were assessed by analyzing the improvement of ascites and serum sodium, changes of other correlative laboratory tests, and survival data from selected studies.

#### The improvement of ascites

**Fig. 2:**
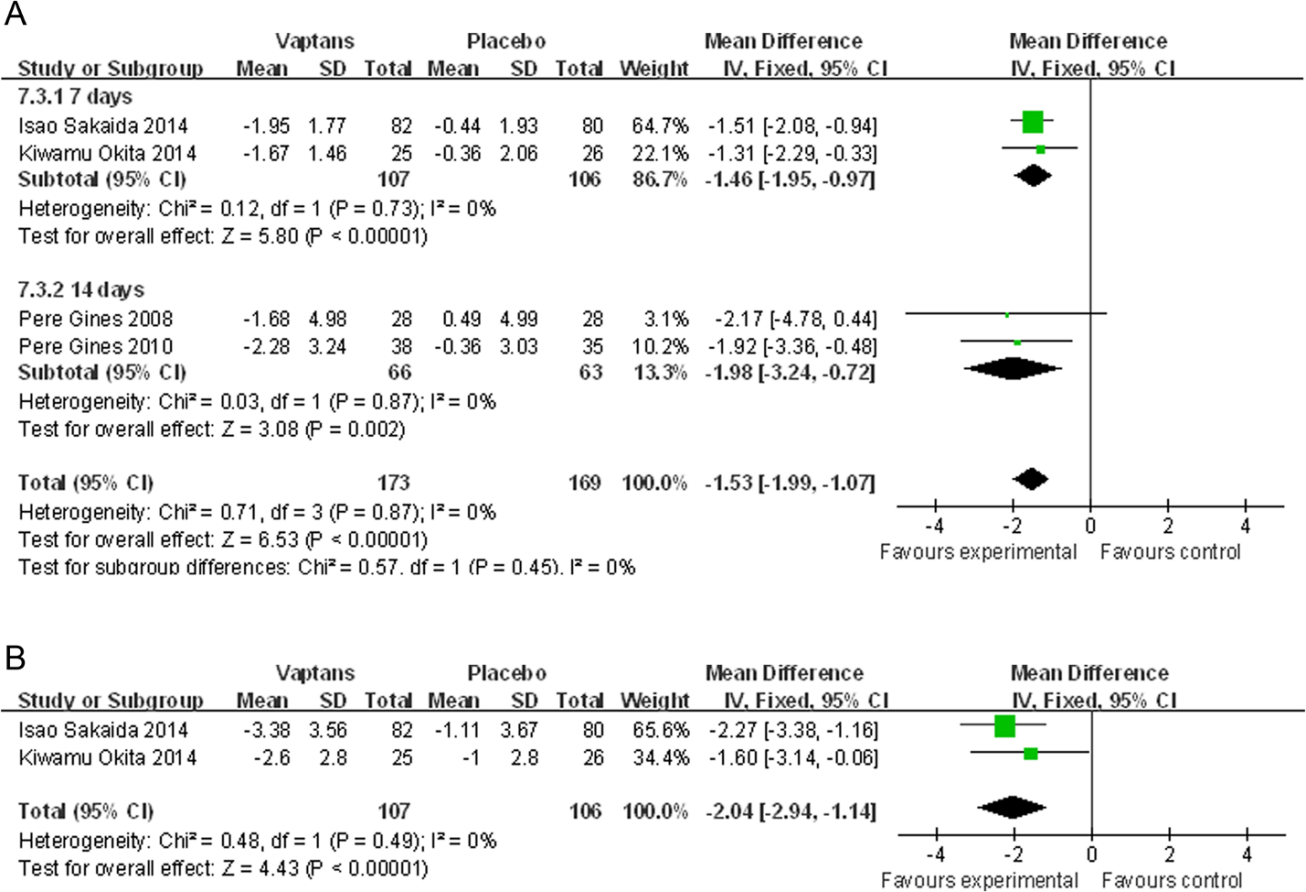
**a** The comparison between the vaptans groups and placebo groups about the mean change of weight after 7-days and 14-days intervention. Studies are identified by the first author’s full name and year of publication. Weighted mean differences are pooled using the fixed effects model. Abbreviation: SD, standard deviation; CI, confidence interval. **b** The comparison between the vaptans groups and placebo groups about the mean change of abdominal girth after 7-days intervention. Studies are identified by the first author’s name and year of publication. Weighted mean differences are pooled using the fixed effects model. Abbreviation: SD, standard deviation; CI, confidence interval

The change of weight and abdominal girth was usually used to assess the effectiveness of the treatment for ascites. The mean change of weight and abdominal girth was measured as the ending data minus the baseline data (more negative represented more decrement). After pooling and analyzing the change of weight, we found patients in vaptans arm reduced more than in placebo arm after 7-days [23, 24] and 14-days [18, 20] both (WMD = −1.46Kg, 95 % CI [−1.95,-0.97], p < 0.00001 and WMD = −1.98Kg, 95 % CI [−3.24,-0.72], p = 0.002, Fig. 2a). Two studies [23, 24] supplied the information of the mean change of abdominal girth after 7 days of intervention, and patients in vaptans arm were found to be more decrement (WMD = −2.04 cm, 95 % CI [−2.94,-1.14], p < 0.00001, Fig. 2b). When we defined ascites worsening as either need for therapeutic paracentesis, increase in diuretic dose or weight gain of > =2 kg during the study period [18, 20], we found the ratio of worsening ascites was lower by the employment of vaptans (RR = 0.51, 95 % CI [0.34, 0.77], p = 0.001).

The obvious reduction of weight and abdominal girth followed the significant effect of promoting renal water excretion of vaptans. Except three studies, which were published as abstract, all of the other studies reported vaptans could significantly increase ascites patients’ 24 h urine output compared with placebo, whether patients in the two controlled groups taking conventional diuretic (spironolactone, or furosemide) or not during studies. This effect appeared from the first dose of vaptans, and it seemed as dose-dependent [14, 15, 17, 18, 20]. That meant within the scope of the adopted dose of vaptans in all studies (lixivaptan/VPA-985 was 25 mg, 50 mg, 100 mg, 200 mg, 300 mg per day, or 100 mg, 200 mg per day; RWJ-351647 was 1 mg, 2 mg, 5 mg per day; satavaptan was 5 mg, 12.5 mg, 25 mg per day), higher dose could bring larger rise of urine.

#### The improvement of serum sodium

**Fig. 3:**
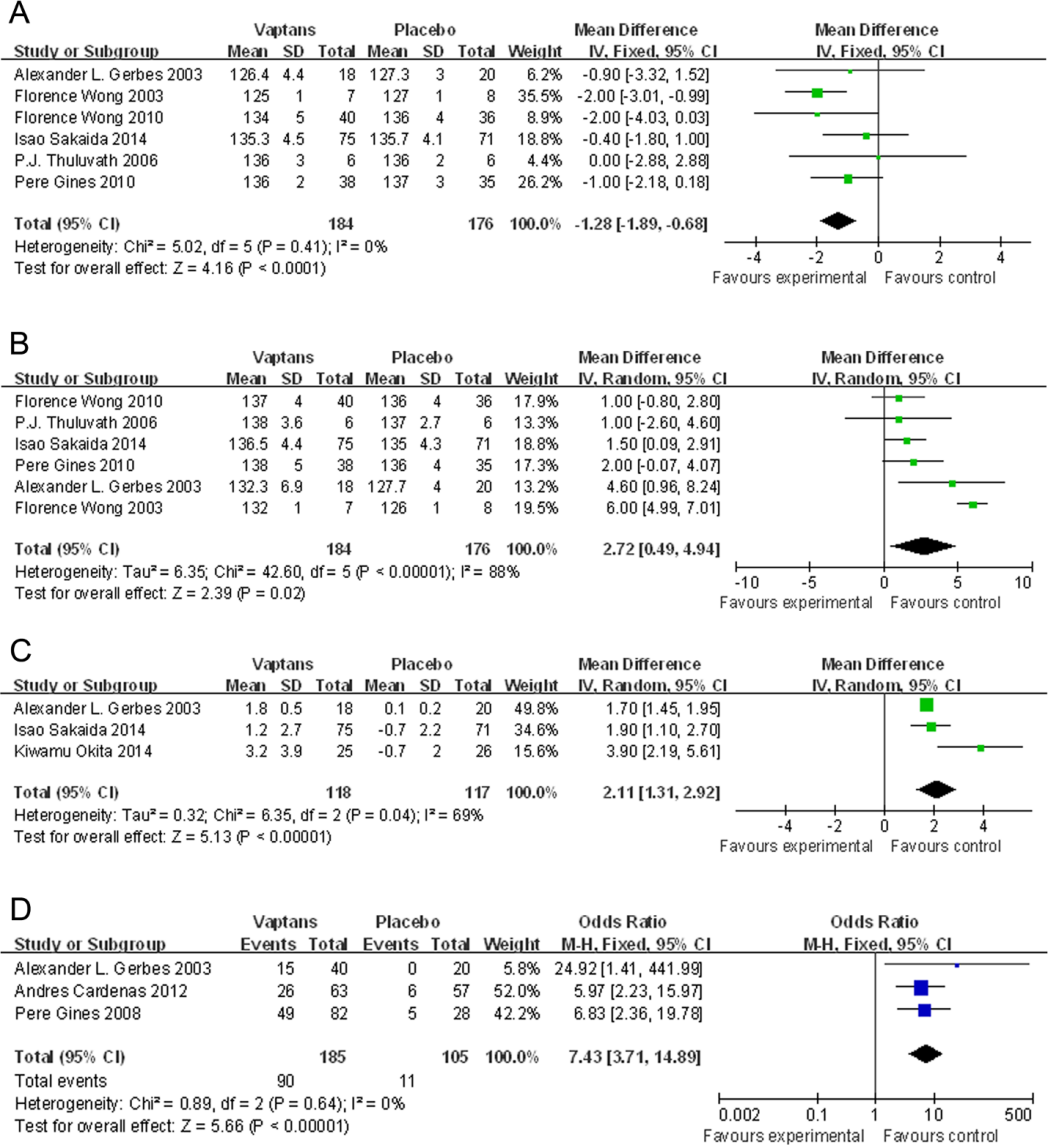
**a** The comparison between the vaptans groups and placebo groups about the mean of baseline serum sodium concentration before interventions. Studies are identified by the first author’s name and year of publication. Weighted mean differences are pooled using the fixed effects model. Abbreviation: SD, standard deviation; CI, confidence interval. **b** The comparison between the vaptans groups and placebo groups about the mean of ending serum sodium concentration after short-term (1-14d) interventions. Studies are identified by the first author’s name and year of publication. Weighted mean differences are pooled using the random effects model. Abbreviation: SD, standard deviation; CI, confidence interval. **c** The comparison between the vaptans groups and placebo groups about the mean change of serum sodium concentration after 7 days interventions. Studies are identified by the first author’s name and year of publication. Weighted mean differences are pooled using the random effects model. Abbreviation: SD, standard deviation; CI, confidence interval. **d** The comparison between the vaptans groups and placebo groups about the normalization ratio of serum sodium concentration within 1 week (4, 5, 7d) interventions. Studies are identified by the first author’s name and year of publication. The Odds Ratios are pooled using the fixed effects model. Abbreviation: CI, confidence interval

Just short-term data (1-14d) about the change of serum sodium concentration of ascites patients could be available according to the extracted information. 6 studies (containing 360 patients) [15–17, 19, 20, 23] reported the baseline and ending serum sodium concentration both, and we separately compared these two groups of data to detect the effects of intervention measures. Even patients in the vaptans arm had a lower baseline of serum sodium concentration than in the control arm with placebo (WMD = −1.28 mmol/L, 95 % CI [−1.89, −0.68], p < 0.0001, Fig. 3a), when comparing the ending data, the patients in vaptans arm had higher serum sodium concentration (WMD = 2.72 mmol/L, 95 % CI [0.49, 4.94], p = 0.02, Fig. 3b). As the heterogeneity was 88 % (>50 %), the random-effects model was used when comparing the ending data. In these 6 studies, most patients had gotten spironolactone or/and furosemide if necessary except the patients in 1 study. This one did not refer to the use of conventional diuretics, and when we removed this 1 exception, the result of analysis was basically the same. So from the outcome of analysis, we found short-term application of vaptans on the basis of using conventional diuretics could more significantly improve the serum sodium concentration of cirrhosis patients with ascites than application of conventional diuretics only.

There were 3 studies [15, 23, 24] offered information of the mean change between baseline and ending serum sodium concentration after using vaptans for 7 days. We got the same conclusion that patients in vaptans arm obtained larger improvement in serum sodium concentration during the studies than patients in the placebo group (WMD = 2.11 mmol/L, 95 % CI [1.31, 2.92], p < 0.00001, Fig. 3c), which meant vaptans could contribute to recover the patients’ serum sodium concentration with ascites.

The normalization ratio of serum sodium (normal value was defined as > =136 mmol/L) after treatment of vaptans within 1 week (4, 5, 7d) were mentioned in 3 studies [15, 18, 21]. The analysis result told that vaptans were significant helpful in normalizing ascites patients’ serum sodium concentration (OR = 7.43, 95 % CI [3.71, 14.89], p < 0.00001, Fig. 3d)

#### Survival

**Fig. 4:**
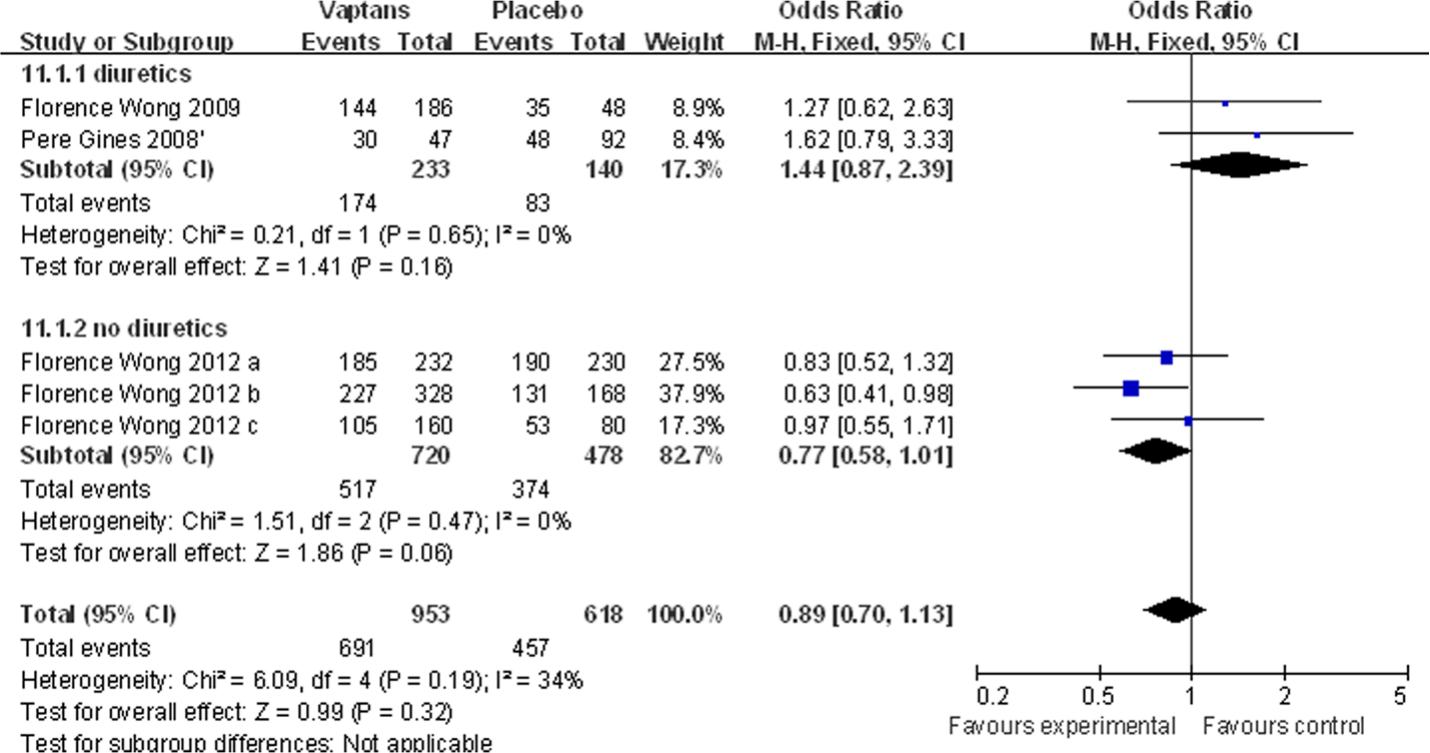
The comparison between the vaptans groups and placebo groups about 1-year survival. Studies are identified by the first author’s name and year of publication. The data of intervention groups would be gathered up as one to make the comparison if there are several dose groups comparing with one control group. The Odds Ratios are pooled using the fixed effects model. Abbreviation: CI, confidence interval

Three studies containing five RCTs (1571 patients) reported the information of 1-year survival after the intervention of vaptans or placebo for cirrhosis patients with ascites [22, 26, 27]. However, the analysis result of survival was inconsistent with the effects of relieving ascites symptoms and elevating serum sodium concentration which were mentioned above. Vaptans did not extend patients’ lifetime comparing to the placebo (OR = 0.89, p = 0.32). In order to eliminate the influence of conventional diuretics (spironolactone or/and furosemide) to vaptans, we established two subgroups according to whether the included studies treated patients (both vaptans and placebo groups) with conventional diuretics or not. It seemed both two subgroups (patients getting conventional diuretics or not), the included vaptans did not extend patients’ lifetime (p = 0.16, and p = 0.06, Fig. 4). There also were 3 studies (419 patients) mentioned the survival after short-term (2, 4, 12 weeks) administration of vaptans, and as the same with long-term survival, no increase or drop of mortality in the vaptans groups was found versus placebo patients in these studies (OR = 1.09, p = 0.85).

### Safety

We assessed the safety of all these studies by analyzing the incidence rate of adverse events, which occurred during researches. Adverse events mainly presented as general events, like thirsty, and serious events, which mostly was the severe manifestations and complications of liver cirrhosis.

**Fig. 5:**
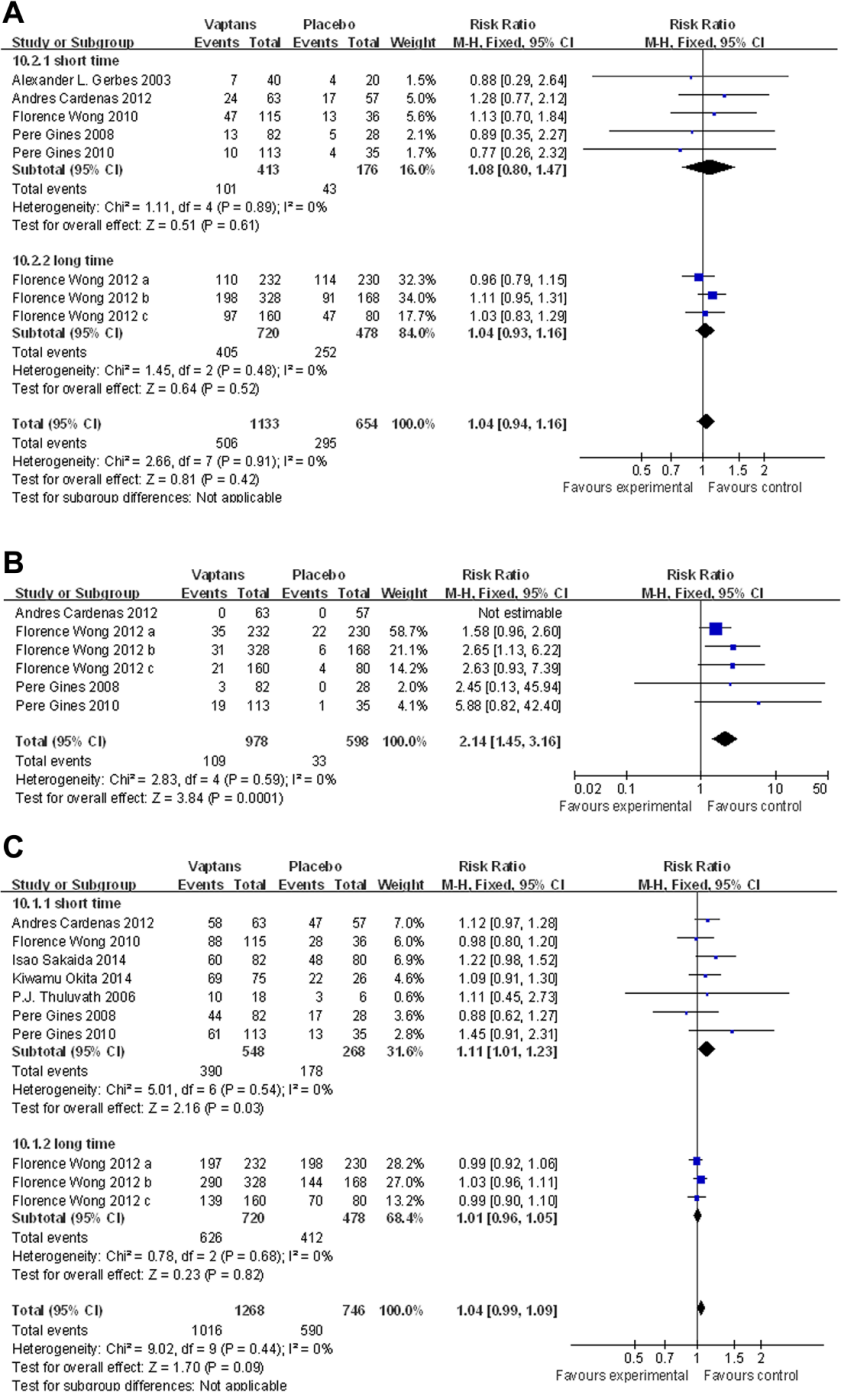
**a** The comparison between the vaptans groups and placebo groups about the total adverse events. Studies are identified by the first author’s name and year of publication. The data of intervention groups would be gathered up as one to make the comparison if there are several dose groups comparing with one control group. The Risk Ratios are pooled using the fixed effects model. Abbreviation: CI, confidence interval. **b** The comparison between the vaptans groups and placebo groups about the serious adverse events. Studies are identified by the first author’s name and year of publication. The data of intervention groups would be gathered up as one to make the comparison if there are several dose groups comparing with one control group. The Risk Ratios are pooled using the fixed effects model. Abbreviation: CI, confidence interval. **c** The comparison between the vaptans groups and placebo groups about the incidence rate of deterioration of liver function events. Studies are identified by the first author’s name and year of publication. The data of intervention groups would be gathered up as one to make the comparison if there are several dose groups comparing with one control group. The Risk Ratios are pooled using the fixed effects model. Abbreviation: CI, confidence interval

Though the incidence of adverse events were light higher in the vaptans groups following short-term (1d-3 months) intervention (RR = 1.11, 95 % CI [1.01, 1.23], p = 0.03), there was no significant difference between the two groups about the total adverse events (RR = 1.04, p = 0.09, Fig. 5a). The occurrence of the total serious events was almost the same between vaptans and placebo arms (RR = 1.04, p = 0.42), no matter the intervention time was short (p = 0.61) or long (p = 0.52, Fig. 5b). More patients would likely feel thirsty after taking vaptans than patients with placebo (RR = 7.02, 95 % CI [3.04, 16.19], p < 0.00001). Excessive correction of serum sodium concentrations (the serum sodium level was >145 mmol/L after the use of vaptans) was more frequently noted in patients under the employment of vaptans (RR = 2.14, 95 % CI [1.45, 3.16], p = 0.0001). Some serious events, which were the complications of liver cirrhosis, like gastrointestinal bleeding (p = 0.09), renal impairment (p = 0.16), hepatic encephalopathy (p = 0.70), emerged in the same between experimental and control patients. We considered hepatic failure, hepatorenal syndrome, veno-occlusive liver disease, hepatic encephalopathy, and the increase of blood bilirubin as the deterioration performances of liver function. There was no significant difference about the incidence rate of deterioration of liver function between the patients who adopted vaptans or not.

Almost all studies found the administration of the included vasopressin V2-receptor antagonist had fewer clinically significant changes of heart rate and blood pressure, which suggested the significant effect of diuresis would not affect the cardiovascular function.

### Sensitivity analysis and Publication bias

**Fig. 6:**
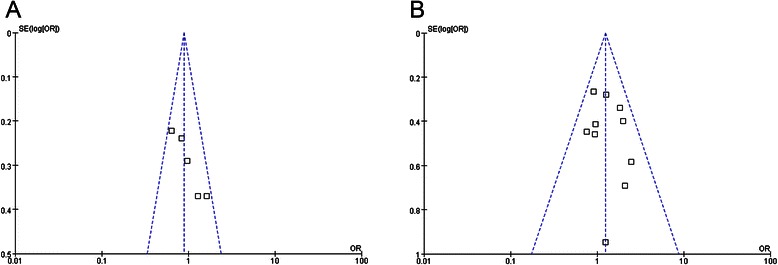
**a** Funnel plots to detect publication biases of survival. **b** Funnel plots to detect publication biases of adverse events

The sensitivity analysis was carried out in every meta-analysis mentioned above by performing the fixed effects model and the random effects model meta-analyses at the same time and observing whether the conclusions differed from each other. And no significant difference was found. When we employed funnel plots to detect publication biases, no significant things was found (taking the analysis of survival and adverse events for example, Fig. 6a, and Fig. 6b).

## Discussion

In this meta-analysis we summarized the efficiency and safety of vasopressin V2-receptor antagonists (one kind of vaptans) in cirrhosis patients with ascites from all related previous studies. vasopressin V2-receptor antagonist could significantly improve the ascites and low serum sodium status (or even hyponatremia) of liver cirrhosis patients. However, no survival benefit was detected, whether with the administration of vaptans for short-term or long-term.

Liver disease is a serious and widespread health problem, especially in Asia, because of the epidemic of hepatitis (chronic hepatitis B, mainly) [28]. For cirrhosis patients with ascites, no matter they are in the waiting list of liver transplantation or could not receive transplantation because of some objective reasons, symptomatic treatment is very important. Vaptans was proved to be a kind of effective aquaretics in SIADH patients [29], heart failure patients [30]. And according to our analyzed data, vaptans can also play a remarkable role on cirrhosis patients in aquaretic effect. Except for patients in three included studies published as abstract, which did not mention the data about the aquaretic effect of satavaptan, all of the other included patients were reported to present an increase of 24 h urine output after the administration of vaptans. And we found this effect did not rely on whether vaptans was employed alone or in combination with conventional diuretics. In some included studies [17, 18], ascites patients were reported to show low or even non response to the use of conventional diuretics at 24 h urine output. However, vaptans still showed significant aquaretic effect. So vaptans may be helpful for refractory ascites patients, who presented low response to conventional diuretics [31], to reduce ascites volume and the need for paracentesis, without the generation of more adverse events by increasing diuretic dose.

Generally, ascites patients had some ascites associated clinical symptoms, such as abdominal distension, loss of appetite, breathing difficulty, and so on. These symptoms may lead to deterioration of quality of life [2]. Because of the significant aquaretic effect of vaptans, patients in vaptans groups presented significant decrease in bodyweight and abdominal girth, which was considered to reflect improvement of the clinical symptoms for ascites patients. And that meant vaptans could significantly improve patients’ quality of life. This improvement is very important for cirrhosis patients [32].

Serum sodium concentration was proved to be an very important prognostic indicator for cirrhosis patients [33]. Low serum sodium concentration frequently appeared on cirrhosis patients with ascites [34]. Some previous studies reported that lower serum sodium concentration, or even hyponatremia, could be associated with lower survival [35]. So the treatment of correcting the serum sodium concentration was necessary for cirrhosis patients. From the selected studies and the analysis results we knew, serum sodium concentration was significantly increased in the vaptans groups, no matter whether the administration of vaptans was in association with conventional diuretics or not. And the increment presented as dose related in some studies. The significant effect of correcting the serum sodium concentration derived from the mechanism of vasopressin V2-receptor antagonists. V2 receptor is expressed principally in main cells of the renal-collecting-duct system, and its activation leads to increased resorption of free water [36]. So vasopressin V2-receptor antagonists may induce a highly hypotonic diuresis without affecting the excretion of electrolytes [7], and that leads to the increase of serum sodium naturally.

Because serum sodium concentration has already been incorporated into the Model for End-Stage Liver Disease (MELD) score, and the MELD is currently used as assessment criteria for patients awaiting liver transplantation [35], the significant increase of serum sodium level would reduce the score and priority of patients in the waiting list inevitably. However, it has been proved that hyponatremia could increase the mortality of perioperative liver transplant [37]. When we just consider transplanting patients with hyponatremia preferentially without correcting the serum sodium actively, it may achieve negative results for reducing the liver transplant mortality [38]. So even if it affects the priority, effective medical management to improve the serum sodium, like the vasopressin V2 receptor antagonists, remains important in cirrhosis patients with hyponatremia. Of course the new and effective aquaretics is also very important for the cirrhosis patients in some countries in which lots patients could not receive timely liver transplantation.

What puzzled us was, though the significant effect on the relief of symptoms and the elevating of serum sodium concentration, patients’ short-term and long-term survival did not change much after the administration of vasopressin V2 receptor antagonists. There were 3 studies containing 1571 patients and 3 studies containing 419 patients respectively reported the information of 1-year survival and short-term (2, 4, 12 weeks) survival. The pooled data did not prove the included vaptans extend patients’ life. In our opinions, the reasons caused the similar outcome of survival for all of the included patients were: 1. The survival data was insufficient as more than a half of included studies (8 studies) did not provide survival data. More survival data, especially long-term survival, was needed. 2. Most included studies did not manage the analysis and researches by stratifying the patients according the severity of liver disease. 3. Most of patients in these RCTs could be categorized as end-stage liver disease patients, and the prognosis were extremely poor for these persons. From the analysis of survival data we could also find that, though it was not significant, vaptans may extend patients’ life time when it was used with conventional diuretic (the OR value was 1.44).

Excessive correction of serum sodium concentrations (>145 mmol/L) was a typical treatment-emergent adverse event caused by vaptans, and it may cause some serious consequence, like osmotic demyelination and myelinolysis [10]. 6 trails reported about 11.1 % patients in the vaptans group emerged excessive correction of serum sodium at least one time [18, 20–22]. The RR value was 2.14 when comparing with placebo group (about 5.5 % patients in placebo group). It was obviously that treatment with higher dose vaptans (lixivaptan/VPA-985 300 mg per day; RWJ-351647 5 mg per day; satavaptan 25 mg per day) meant higher chance of excessive correction of serum sodium for cirrhosis patients with ascites. So the dosage must be good controlled and the change of serum sodium concentration should be monitored carefully when we treat cirrhosis patients with vaptans clinically. However, no patients in these 6 trails retreated from studies and no serious consequences happened because of the emerging of excessive of correction of serum sodium concentrations. The liver injury was regarded as another limit of the vaptan’s clinical application. However, from the analytic work we found the employment of vaptans would not lead to deterioration of liver function in cirrhosis patients with ascites. Although the difference was not significant, RR value was 0.88 might indicate the incidence rate of deterioration of liver function may be even smaller for the patients who used vaptans. To some extent, the vasopressin V2 receptor antagonists were safe in the clinical application.

## Conclusions

From our analysis we drew a conclusion that, as a kind of new and effective aquaretics, vasopressin V2 receptor antagonists could play an effective role in elevating serum sodium concentration and symptomatic treatment for cirrhosis patients with ascites, especially for refractory ascites patients who presented insufficient response to conventional diuretics. Necessary monitoring must be adopted when use these vaptans in the clinical in order to avoid the happening of treatment-emergent adverse events (like excessive of correction of serum sodium). Though the analysis of the included studies did not find evidence of vasopressin V2 receptor antagonists extending cirrhosis patients’ lifetime, we could not ignore this effect easily considering the remarkable effects of elevating serum sodium concentration and improvement of ascites. Under ideal conditions, more RCTs of each vaptans focusing on the survival data may be needed.
